# Diversity and sex differences in rectal gland volatiles of Queensland fruit fly, *Bactrocera tryoni* (Diptera: Tephritidae)

**DOI:** 10.1371/journal.pone.0273210

**Published:** 2022-08-24

**Authors:** Cynthia Castro-Vargas, Gunjan Pandey, Heng Lin Yeap, Michael J. Lacey, Siu Fai Lee, Soo J. Park, Phillip W. Taylor, John G. Oakeshott

**Affiliations:** 1 Land and Water, Commonwealth Scientific and Industrial Research Organisation, Black Mountain, ACT, Australia; 2 Applied BioSciences, Macquarie University, North Ryde, NSW, Australia; 3 Australian Research Council Centre for Fruit Fly Biosecurity Innovation, Macquarie University, North Ryde, NSW, Australia; 4 Bio21 Molecular Science and Biotechnology Institute, University of Melbourne, Parkville, VIC, Australia; 5 National Collections and Marine Infrastructure, Commonwealth Scientific and Industrial Research Organisation, Black Mountain, ACT, Australia; University of Thessaly School of Agricultural Sciences, GREECE

## Abstract

Rectal gland volatiles are key mediators of sexual interactions in tephritid fruit flies. We used solid-phase microextraction (SPME) plus gas chromatography-mass spectrometry (GC-MS) and gas chromatography-flame ionization detection (GC-FID) to substantially expand rectal gland chemical characterisation of the Queensland fruit fly (*Bactrocera tryoni* (Diptera: Tephritidae); Qfly). The SPME GC-MS analysis identified 24 of the 30 compounds previously recorded from Qfly rectal glands, plus another 21 compounds that had not previously been reported. A few amides and fatty acid esters dominated the chromatograms of males and females respectively, but we also found other esters, alcohols and aldehydes and a ketone. The GC-FID analyses also revealed over 150 others, as yet unidentified, volatiles, generally in lesser amounts. The GC-FID analyses also showed 49 and 12 compounds were male- and female-specific, respectively, both in single sex (virgin) and mixed sex (mostly mated) groups. Another ten compounds were male-specific among virgins but undetected in mixed sex groups, and 29 were undetected in virgins but male-specific in mixed sex groups. The corresponding figures for females were four and zero, respectively. Most short retention time peaks (including a ketone and an ester) were male-specific, whereas most female-biased peaks (including five fatty acid esters) had long retention times. Our results indicate previously unsuspected diversity of rectal gland volatiles that might have pheromone functions in males, but far fewer in females.

## Introduction

It is widely accepted that differences in sex pheromone signalling systems can serve as effective pre-mating isolating mechanisms between closely related insect species [1–5], and in a few well-studied sibling species of Lepidoptera and Coleoptera these differences have been traced to isomeric differences in a single pheromonal compound [6, 7]. There is mounting evidence that many aspects of the courtship and mating behaviours of true fruit flies (Diptera: Tephritidae) also involve pheromones [reviewed in 8], and it may be that differences in these pheromones also inhibit hybridization among the many sympatric species in this highly speciose group (>5,000 described species [9]). In most tephritids, the focus has been on volatiles released by males [10–15], but female-produced volatiles have also been found to be important in some cases [16–18], and in some species compounds emitted by both sexes are important mediators of mating [19–21]. Volatiles emitted from a specialised rectal gland complex have commonly been implicated as key to chemically mediated sexual interactions [reviewed in 8, 22], but little is known about the specific compounds involved, and the evidence available implicates the action of multi-component blends.

The best characterised tephritid pheromone system to date involves the Mediterranean fruit fly, *Ceratitis capitata*. A complex mix of at least 69 male rectal gland volatiles, including a variety of carboxylic acids, esters, alcohols and sesquiterpenes, has been identified in this species [13, 23–34]. Synthetic mixtures of a few of these compounds have been found to attract both males and females in laboratory trials [35–37], as well as in the field [32, 37]. Many of these compounds individually elicit electroantennogram (EAG) responses, although the responses to several compounds differ between males and females [25, 27, 31]. There is little relationship between the amount and EAG bioactivity of the compounds tested [27], but five of the most abundant compounds (ethyl acetate, geranyl acetate, ethyl (*E*)-3-octenoate, (*E*, *E*)-α-farnesene and 1-pyrroline) have been individually found to attract females in a laboratory flight tunnel [36].

The evidence available for other tephritid genera also implicates multiple rectal gland volatiles in sex pheromone activity. The male pheromone from the Mexican fruit fly, *Anastrepha ludens*, consists of a blend of at least seven lactones and unsaturated alcohols, which attract virgin females, stimulate searching activity, and increase female-female agonism [11, 38–41]. In the oriental fruit fly, *Bactrocera dorsalis*, male rectal gland volatiles consist mainly of long-chain fatty acids as well as diverse spiroacetals, although it is not known which of these compounds contribute to sex pheromone functions [10, 20, 42–44].

Work to date on the Queensland fruit fly (Qfly), *Bactrocera tryoni*, also suggests a complex sex pheromone system. Courtship and initiation of mating in this species occur during a brief window of about 30 minutes at dusk [45, 46] when sexually mature males release a blend of volatiles that has been synthesised and stored in their rectal glands [47, 48]. The blend is dispersed by rapidly drawing the wings backwards and forwards (‘calling’), which also produces an audible buzzing sound [49, 50]. Under some laboratory conditions at least, sexually mature females are shown to respond to crushed male rectal glands [48, 51], suggesting that the glands indeed contain compounds which could act as male sex pheromones in this species.

Gas-chromatography-mass spectrometry (GC-MS) analyses of organic phase extracts of male Qfly rectal glands have identified six prominent aliphatic amides, specifically *N*-(2-methylbutyl)acetamide, *N*-(3-methylbutyl)acetamide, *N*-(2-methylbutyl)propanamide, *N*-(3-methylbutyl)propanamide, *N*-(2-methylbutyl)-2-methylpropanamide and *N*-(3-methylbutyl)-2-methylpropanamide, with the *N*-(3-methylbutyl)propanamide clearly most abundant as judged by their relative peak areas [52, 53]. This amide blend has been proposed to function as a short-range male sex pheromone [45, 52, 54]. However, empirical evidence for pheromonal function for these particular compounds is scant, and Bellas & Fletcher [52] note that the amides do not have the sweet scent associated with calling males, so at least some of the pheromone function may be due to other less abundant volatiles for which the females may have lower odour perception thresholds.

More recent GC-MS analyses by Noushini et al. [55] have also found six volatile esters, ethyl 2-methylpropanoate, ethyl propanoate, ethyl 2-methylbutanoate, *n*-propyl 2-methylpropanoate, ethyl 2-methylpentanoate and diethyl succinate, in the organic phase extracts of male Qfly rectal glands. Notably, some of these esters are also produced by many ripe fruits [56–59], and some act as attractants to Qflies of both sexes [60]. Although the esters must have been endogenously produced in the flies studied by Noushini et al. [55], there are other cases where compounds ingested by tephritids have been translocated to their rectal glands via the haemolymph [48, 61–64]. Two well-studied examples of this involve 4-(4-acetoxyphenyl)-2-butanone and 4-(4-hydroxyphenyl)-2-butanone (a.k.a. cuelure and raspberry ketone, respectively), which are plant polypropanoids [65, 66]. Cuelure is a strong attractant for male Qflies (as is raspberry ketone) and is widely used as a lure in traps also containing insecticide for monitoring and population suppression [67–70]. When ingested, cuelure is transported through the haemolymph to the rectal gland, where it is stored in its hydrolysed form as raspberry ketone, while ingested raspberry ketone is transported and stored in unaltered form [62, 71].

Twenty two compounds have also been reported in a GC-MS analysis of the organic phase extracts of rectal glands from female Qflies [55, 72, 73]. These include five of the amides found in male rectal gland extracts (see above), plus six spiroacetals and six saturated and five unsaturated long-chain esters (hereafter fatty acid esters). Unlike the situation in male rectal glands, where the amides predominate, the esters were found to be the most abundant compounds in female rectal glands. Nothing has yet been published on the behavioural effects of these compounds.

In light of the very limited evidence that the abundant amides are responsible for much of the behavioural effects of the volatiles emitted from male Qfly rectal glands, plus the evidence from *C*. *capitata* that abundance does not correlate well with behavioural activity, the aims of the current study are to build a more comprehensive inventory of the volatiles produced by Qfly rectal glands and to determine how they differ between the sexes and with mating history. We first use solid-phase microextraction (SPME) from the head space of dissected rectal glands plus gas chromatography-mass spectrometry (GC-MS) to substantially expand the range of rectal gland volatiles that have been identified. We then analyse hexane extracts of the glands by gas chromatography-flame ionisation detection (GC-FID) to further broaden the range of volatiles that could be detected and enable quantitative comparisons of their relative abundances across sexes and mating history categories.

## Results

### SPME GC-MS profiles

**Fig 1:**
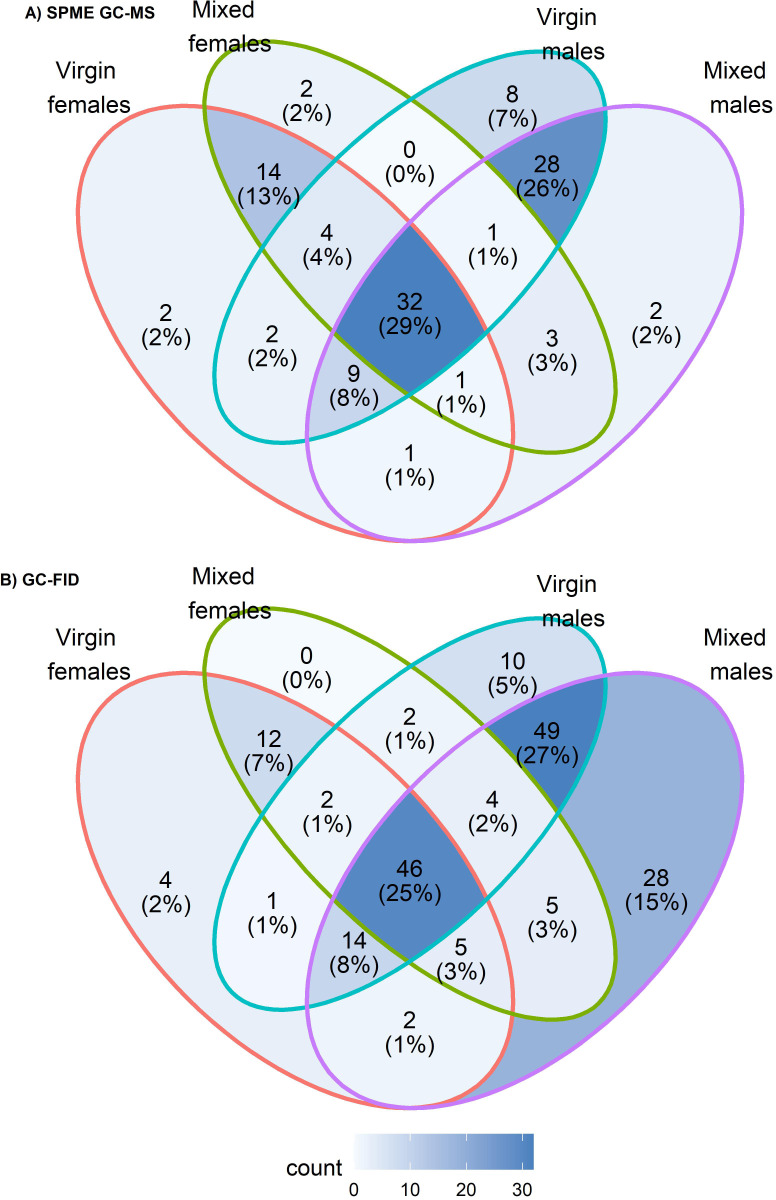
Venn diagrams showing the numbers of peaks detected in each sex/mating history category in the SPME GC-MS (A) and GC-FID analyses (B).

We detected a total of 109 peaks by SPME GC-MS analysis (operating in full scanning mode) of rectal gland contents of sexually mature adults of the S06 strain aged 15–20 days since emergence (Fig 1A, S1 and S2 Figs). Ninety-one peaks were seen in males and 71 in females, with over half the total seen in both sexes. Most of the peaks in each sex were found in flies held in both virgin and mixed sex groups (90–95% of the females in mixed sex groups were assumed to have mated [74]), but several were also specific to one or the other of these categories. Forty-five of the peaks were tentatively identified by analysis of m/z values of fragment ions, supplemented in most cases by comparisons of their Kovats Indices (KIs) to published values, and the identities of 26 of these could then be confirmed against authentic standards (Table 1). The authenticated peaks included all the major peaks in each sex and most of the peaks previously identified in Qfly rectal glands [48, 52, 55, 72, 73, 75] (S1 Table). The major exceptions in the latter were five spiroacetals; we only sporadically observed peaks in the chromatograms where we expected these spiroacetals to elute, and we did not have authentic standards to confirm them. Noushini et al. [55] also found considerable variability in their ability to detect the spiroacetals. Twenty-one of our identified peaks had not been reported in the previous studies, but in general terms our data confirmed both the prominence of the six previously identified aliphatic amides, particularly *N*-(3-methylbutyl)propanamide, in the male profiles, and the prevalence of several methyl and ethyl fatty acid esters in the female profiles.

**Table 1:** Forty-five chemicals found in virgin and mixed sex groups of male (M) and female (F) rectal glands.

No.	Compound	Basis of assignment	Virgin	Mixed	KI observed	KI literature
1	Ethyl propanoate	Auth. Std.	M	M	702	710
2	Ethyl 2-methylpropanoate	Auth. Std.	M	M	752	756
3	(*D*, *L*)- 2,3-Butanediol ^#^	Auth. Std.	n.d.	M F	786	788
4	(*meso*)- 2,3-Butanediol ^#^	Auth. Std.	n.d.	M F	789	788
5	Ethyl 2-methylbutanoate	Auth. Std.	M F	M	843	849
6	*n*-Propyl 2-methylpropanoate	Auth. Std.	M	M	848	852
7	4-Heptanone ^#^	Auth. Std.	M	M	867	872
8	*x*-Octenal^#^ (isomer 1)	Man. Ass.	M	M	902	N/A
9	Ethyl-2-methylpentanoate	Auth. Std.	M	M	933	941
10	*x*-Octenal^#^ (isomer 2)	Man. Ass.	M	M	939	N/A
11	*x*-Octen-1-ol ^#^	Man. Ass.	F	F	1019	N/A
12	2-Ethyl-1-hexanol ^#^	Auth. Std.	M F	M F	1026	1030
13	*N*-(2-Methylpropyl)propanamide^#^	Man. Ass. & KI	M	M	1094	1091
14	*N*-(2-Methylbutyl)acetamide	Auth. Std.	M F	M F	1132	1131
15	*N*-(3-Methylbutyl)acetamide	Auth. Std.	M F	M F	1142	1150
16	(*E*,*E*)-2,8-Dimethyl-1,7-dioxaspiro[5.5]undecane	Man. Ass. & KI	M F	M F	1159	1148
17	2-Bornanone ^#^	Auth. Std.	M	n.d.	1171	1171
18	Diethyl succinate	Auth. Std.	M	M	1182	1182
19	*N*-(2-Methylbutyl)propanamide	Auth. Std.	M F	M F	1201	1198
20	*N*-(3-Methylbutyl)propanamide	Auth. Std.	M F	M F	1212	1204
21	*N-*(*n-*Pentyl)propanamide^#^	Man. Ass.	M F	M F	1221	N/A
22	*N-*(*n*-Pentyl)butanamide ^#^	Man. Ass.	M	n.d.	1232	N/A
23	*N*-(2-Methylbutyl)-2-methylpropanamide	Auth. Std.	M F	M F	1235	1226
24	*N*-(3-Methylbutyl)-2-methylpropanamide	Auth. Std.	M F	M F	1239	1230
25	Methyl dodecanoate	Auth. Std.	F	F	1525	1526
26	3-Methylpentadecane ^#^	Man. Ass. & KI	M	M	1566	1566
27	Ethyl (*Z*)-9-dodecenoate ^#^	Man. Ass. & KI	M F	M F	1583	1595
28	Ethyl dodecanoate	Auth. Std.	M F	M F	1592	1596
29	*n*-Hexadecane ^#^	Man. Ass. & KI	M	M	1598	1600
30	*n*-Propyl dodecanoate	Man. Ass. & KI	F	F	1675	1685
31	Methyl (*Z*)-9-tetradecenoate ^#^	Man. Ass. & KI	M F	F	1716	1703
32	Methyl tetradecanoate	Auth. Std.	M F	M F	1723	1725
33	2-Methylpropyl dodecanoate ^#^	Man. Ass. & KI	F	F	1750	1745
34	Ethyl (*E*)-9-tetradecenoate ^#^	Man. Ass.	F	F	1769	1769
35	Ethyl (*Z*)-9-tetradecenoate	Auth. Std.	M F	M F	1783	1778
36	Ethyl tetradecanoate	Auth. Std.	M F	M F	1788	1795
37	Ethyl 12-methyltetradecanoate ^#^	Man. Ass.	F	F	1868	1861
38	*n*-Propyl tetradecanoate ^#^	Man. Ass.	F	F	1889	1887
39	Methyl (*Z*)-9-hexadecenoate	Man. Ass. & KI	F	F	1911	1911
40	Methyl hexadecanoate ^#^	Man. Ass. & KI	F	F	1928	1927
41	Ethyl (*Z*)-9-hexadecenoate	Auth. Std.	F	F	1981	1975
42	Ethyl hexadecanoate	Auth. Std.	F	F	1989	1990
43	Ethyl 14-methylhexadecanoate ^#^	Man. Ass. & KI	F	F	2011	2013
44	Ethyl (*Z*)-9-octadecenoate	Auth. Std.	F	F	-	2168
45	Ethyl (*E*)-9-octadecenoate	Auth. Std.	F	F	-	2174

Chemicals not previously identified in Qfly rectal glands are indicated with ^#^ and the prefix “*x*” indicates cases of uncertain isomer configurations. The identities of 26 compounds were authenticated by retention time matching with authentic standards (Auth. Std.) in the GC-MS traces. Nineteen other compounds were tentatively identified from their Kovats Indices (KI), and manual assignments (Man. Ass.) using a comparison of their mass spectra with those recorded in the National Institute of Standards and Technology (NIST) mass spectral library (see also S2 Fig). KI values for two compounds over C20 (44 and 45) were not obtained due to unavailability of C21 standards. “N/A” designates the absence of KI information from the literature. Chemicals that were not detected in a particular sex/mating history combination are indicated with “n.d.”.

As well as the six amides, the 20 authenticated compounds (*i*.*e*., Auth. Std. in Table 1) in male rectal glands from virgin and mixed sex groups included five short-chain esters and the diester diethyl succinate, which had also been reported previously in male rectal glands, plus three fatty acid esters only previously reported in female rectal glands [55, 73] and five new compounds, comprising a ketone, terpenoid, and three alcohols. The 11 tentatively identified compounds (*i*.*e*., those for which authentic standards were unavailable) in the male extracts included three additional aliphatic amides, three additional fatty acid esters, two aliphatic aldehydes, two alkanes and a spiroacetal. None of these tentatively identified chemicals have been identified previously in male Qfly rectal glands, although the spiroacetal had been reported in female rectal glands of this species [55, 72, 73].

The 19 authenticated compounds in female rectal glands included the six amides, three alcohols, one short-chain ester and three fatty acid esters that were authenticated in the male samples, plus six additional fatty acid esters (Table 1). All these compounds except the three alcohols have been previously reported in female Qfly rectal glands [55, 73]. The 13 tentatively identified compounds in female rectal glands comprised one amide, four fatty acid esters that had all also been tentatively identified in the male samples, plus six additional fatty acid esters, a spiroacetal and one additional alcohol. Only three of these 13, (*E*,*E*)-2,8-dimethyl-1,7-dioxaspiro[5.5]undecane, *n*-propyl dodecanoate and methyl (*Z*)-9-hexadecenoate, had previously been identified in female Qfly rectal glands [55, 72, 73], but four of the fatty acid esters (ethyl (*E*)-9-tetradecenoate, *n*-propyl tetradecanoate, methyl hexadecanoate and ethyl 14-methylhexadecanoate) have been found in cuticular washes of mature adult female Qflies [76]. Our data therefore support the contention of Park et al. that some cuticular chemicals could be produced in Qfly rectal glands and subsequently distributed over their wings and bodies during grooming [76].

In sum, of the 45 authenticated and tentatively identified compounds across the two sexes, 13 are male-specific, 14 are female-specific and 18 were found in both sexes. The male-specific compounds included four short-chain esters, one ketone, two aliphatic aldehydes, two amides, two long-chain alkanes, the diester diethyl succinate and the terpene 2-bornanone, but no fatty acid esters. By contrast, all but one of the female-specific compounds were fatty acid esters, the exception being a long-chain alcohol. The 18 compounds common to both sexes included seven long-chain esters but notably also seven amides, as well as two short-chain alcohols, a short-chain ester and a long-chain alcohol.

Six of the 45 identified compounds showed qualitative differences between the males held as virgins and those held in mixed sex groups (Table 1). *N-(n*-pentyl)butanamide and 2-bornanone were only detected in males held as virgins, and the two 2,3-butanediol isomers were only detected in flies of both sexes held in mixed sex groups. Two other compounds were detected in flies of both sexes as virgins but only one sex in mixed sex groups; ethyl 2-methylbutanoate in males of mixed sex groups and methyl (*Z*)-9-tetradecenoate in females of mixed sex groups.

### GC-FID profiles

GC-FID analysis of rectal gland samples from flies of both sexes kept as virgin or in mixed sex groups showed up to 215 peaks with retention time (Rt) values from about 4.6 to 21 min. Only 184 of the peaks that were present in at least half the samples in at least one of the four sex/mating history categories were retained for further analysis (Table 2, Fig 1B, S1 Dataset, S2 and S3 Tables, S3 Fig). Nevertheless, this number is actually about 69% higher than the number of volatile compounds that we recovered from the SPME GC-MS analysis, likely due in large part to the different extraction methods (hexane extraction and SPME respectively for the GC-FID and GC-MS analyses [55, 77, 78], but also in some measure to the differences in the detectors [79–81]. Overall, however, the proportions of peaks in the different sex and mating history categories were very similar in the two data sets (Fig 1).

**Table 2:** Numbers of GC-FID peaks detected classified by sex specificity/ selectivity (F = female, M = male), Rt range and abundance category.

Type	Virgin	Mixed	Number of peaks		
F-biased (34)	F-specific (21)	F-specific (12)	KI range 848–1130	KI range 848–1130	KI range 848–1130
			0, 0, 1	0, 0, 1	2, 5, 3
					**(Ethyl tetradecanoate, 20%)**
		F-selective (3)	-	-	1, 2, 0
					**(Ethyl (*E*)-9-octadecenoate, 5%)**
		~ (2)	-	-	0, 1, 1
		n.d. (4)	0, 1, 0	-	0, 1, 2
	F-selective (10)	F-selective (7)	-	1, 0, 0	**6, 0, 0**
					**(Ethyl (*Z*)-9-hexadecenoate, 23%)**
		~ (2)	-	-	**2, 0, 0**
					**(Ethyl (*Z*)-9-tetradecenoate, 10%)**
		n.d. (1)	-	-	0, 1, 0
	~ (3)	F-specific (2)	-	-	0, 2, 0
		F-selective (1)	-	-	0, 1, 0
					(Methyl tetradecanoate)
M-biased (110)	M-specific (63)	M-specific (49)	0, 6, 4	1, 1, 1	1, 11, 24
			(*n*-propyl 2-methylpropanoate)	**(*N*-(3-Methylbutyl)-2-methylpropanamide, 5%)**	
		n.d. (10)	0, 0, 1	1, 0, 0	0, 1, 7
		~ (4)	-	-	0, 0, 4
	M-selective (4)	M-specific (2)	-	0, 2, 0	-
				(*N*-(2-Methylbutyl)acetamide)	
		M-selective (2)	-	2, 0, 0	-
				**(*N*-(3-Methylbutyl)acetamide, 9%)**	
				**(*N*-(3-Methylbutyl)propanamide, 67%)**	
	~ (14)	M-specific (12)	0, 0, 3 (4-Heptanone^#^)	0, 0, 1	1, 2, 5
		M-selective (2)	-	1, 0, 0	1, 0, 0
				**(*N*-(2-Methylbutyl)propanamide, 4%)**	
	n.d. (29)	M-specific (29)	0, 1, 4	0, 1, 4	0, 4, 15
				(Diethyl succinate)	
Changed specificity (4)	M-specific (2)	F-specific (2)	-	-	0, 2, 0
	F-specific (2)	M-specific (2)	-	-	0, 0, 2
No sex bias (36)	~ (31)	~ (31)	0, 4, 0	1, 0, 0	1, 22, 3
			(2-Ethyl-1-hexanol^#^)	**(*N*-(2-Methylbutyl)-2-methylpropanamide, 2%)**	(Methyl dodecanoate)
					(Ethyl dodecanoate)
	n.d. (5)	~ (5)	-	-	0, 4, 1

The numbers in brackets in the three left-hand columns are the total numbers of peaks in the respective sex/mating history categories. The three numbers in each cell in the three right-hand columns are the numbers of peaks classified as major, intermediate and minor in abundance, respectively. The names of identified peaks in those cells are given in brackets and are bolded, underlined or in plain text if major, intermediate or minor in abundance respectively in the sex/mating history combination in which they were most abundant. For the major identified peaks, their % of summed peak area in the sex/mating history combination in which they were most abundant is also given in the brackets. “~” refers to peaks with no sex specificity or selectivity. Within each mating history category, sex-specific peaks were only detected in one sex; sex-selective peaks showed > log_10_ differences in average peak areas between the sexes; and those with no strong sex bias showed < log_10_ differences in average peak areas between the sexes. “n.d.” refers to peaks not detected in the corresponding category. “^#^”refers to peaks not identified in Qfly rectal glands in previous studies.

Despite the larger numbers overall, the 184 GC-FID peaks retained only included 17 of the 45 compounds that had been identified in the SPME GC-MS analyses. These 17 could be matched to their authentic standards in both analyses and included the six major amides, six fatty acid esters, one short-chain ester, the diester diethyl succinate, one alcohol and a ketone found in the SPME GC-MS data set for males, and five of the amides, seven of the fatty acid esters, the alcohol and ketone in the SPME GC-MS data set for females. Only the alcohol and ketone had not been detected in previous studies.

The short-chain alcohols, esters, aldehydes and ketone all eluted in the Rt range 4.60–10.39 min; the amides and the diester diethyl succinate eluted in the Rt range 10.40–13.59 min; and the fatty acid esters all eluted in the range 13.6–21.0 min. These Rt ranges in our data are approximately equivalent to KI ranges of 840–1095, 1130–1240 and 1525–2175, respectively. Combined across the identified and unidentified peaks, these three Rt ranges, (hereafter the short-, mid- and long-Rt ranges respectively) contained 25, 18 and 141 compounds respectively. Given that 19 of the 21 compounds identified in the equivalent KI range in the SPME GC-MS analysis, and all eight of those identified in the long-Rt range in the GC-FID analysis, were fatty acid esters, many of the 133 unidentified compounds in the latter range could be fatty acid esters as well.

Each peak was classified according to its relative abundance as major, intermediate or minor in each of the four sex/mating history categories if on average across all samples in that category it contributed ≥ 1.0%, 0.10–0.99%, or < 0.10%, respectively, of total peak area in that category (S2 and S3 Tables). Twenty-one of them were classified as major in at least one category, 75 of the others were classified as intermediate in at least one category and the remaining 88 were never more than minor in any category. The profiles in both male mating history categories were dominated by one of the male-biased, mid-Rt amides, *N*-(3-methylbutyl)propanamide, at 55–67% of summed peak areas. Only four and two other peaks in the mid- and long-Rt ranges, respectively, were above 10% of summed peak area in either male category. The four mid-Rt range peaks comprised three amides, and one unidentified peak, while the two long-Rt range peaks were both unidentified (S2 Table). The female profiles were more evenly distributed, with no peak above 30% summed peak area in either female category. The female-biased, long-Rt fatty acid esters ethyl tetradecanoate and ethyl (*Z*)-9-hexadecenoate and two unidentified peaks in the long-Rt range were, however, above 10% of summed peak areas in one or other of the two female categories (S2 Table). Most of the peaks in the long-Rt range in both sexes were minor (67) or intermediate (59) in abundance.

**Fig 2:**
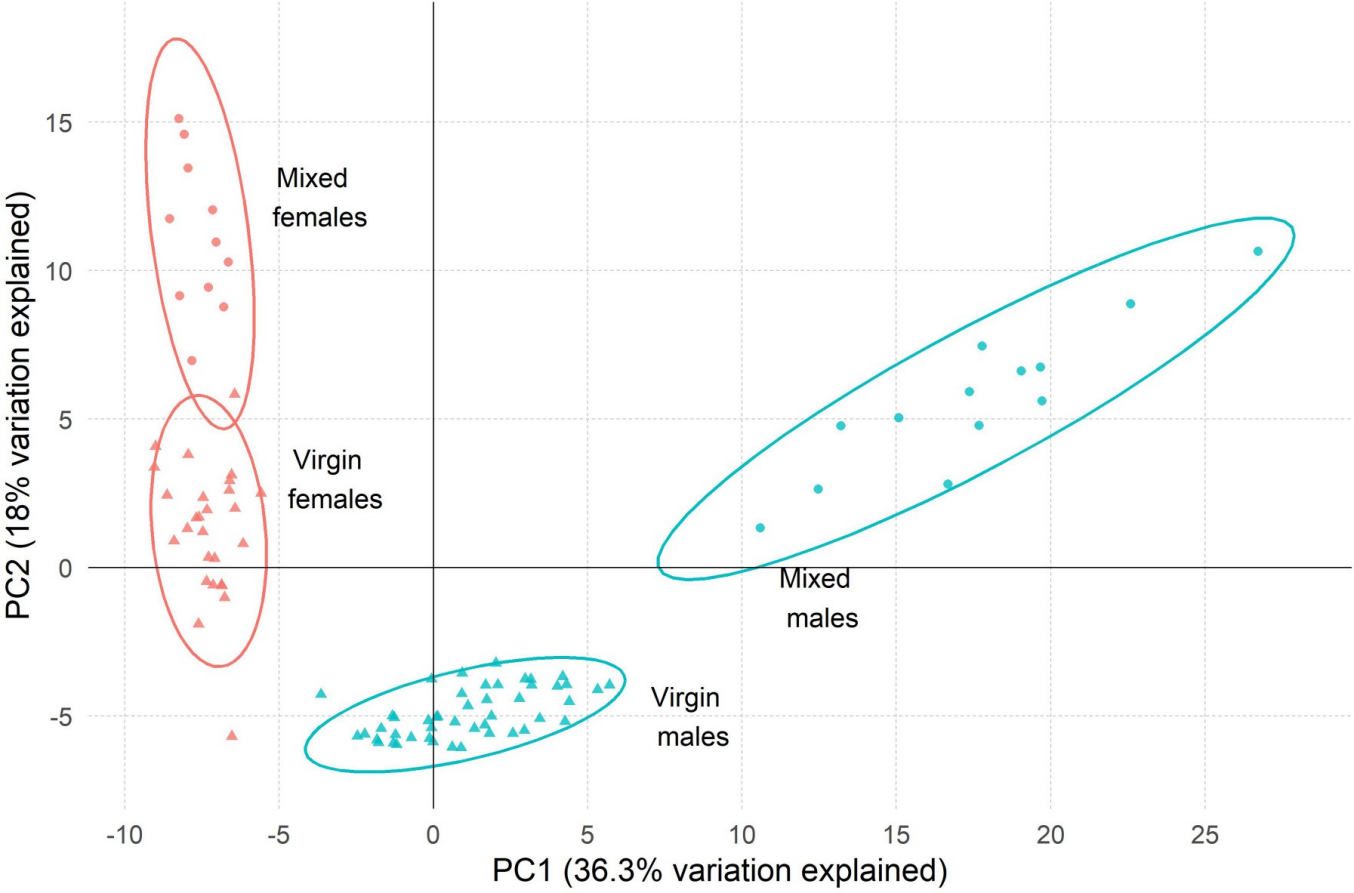
Principal component analysis (PCA) of peaks detected by GC-FID. Samples of the two sexes and mating history categories are distinguished by colour and symbol shape, respectively.

Principal component analysis (PCA) of the relative abundance data for the 184 peaks clearly separated the two sexes on a combination of PC1 and PC2 (explaining 36.3 and 18.0% of the variance, respectively; Fig 2). The samples from mixed sex groups were more dispersed than those from virgins but were clearly separated from the virgins in both sexes. To investigate these sex and mating history effects further, we then grouped the peaks according to the degree of their sex bias, *i*.*e*., as sex-specific (only present in one sex), sex-selective (> log_10_ difference in average peak area between the sexes) or no strong sex bias (< log_10_ difference in average peak area between the sexes) within each mating history category (Table 2).

Even more so than the smaller sample of the 45 identified SPME GC-MS compounds, the GC-FID peaks showed extensive sex bias and in many cases sex specificity. Thus, about 80% of the GC-FID peaks showed some sex bias in their abundance. However, whereas the numbers of male- and female-specific peaks were similar in the SPME GC-MS data set (13 and 14, respectively), the bias in the GC-FID data strongly favoured males. A total of 110 of the latter were male-biased, 88 of them male-specific, whereas far fewer, 34, were female-biased, and just 16 of them female-specific. Given the much larger sample size, the pattern in the GC-FID data may be more representative. As might be expected, the male-biased GC-FID peaks did include five of the amides identified in the SPME GC-MS dataset, but also encompassed 76 long-Rt peaks, most of them minor and some intermediate in abundance. All but four of the female-biased peaks were also in the long-Rt range, five of them identified as fatty acid esters and most of them either major or intermediate in abundance.

Also reminiscent of the SPME GC-MS data, the detection of a significant minority of the GC-FID peaks was both sex- and mating history category-dependent. Thus, ten of the male specific GC-FID peaks were only detectable in virgins and 29 were only detectable in flies from mixed sex groups. By comparison, four of the female-specific peaks were only detected in virgins and none only detected in flies from mixed sex groups. Thus, a relatively small number of peaks specific to either sex were only expressed in virgins and none of the female-specific peaks were only expressed in mixed sex groups, whereas a relatively large number of male-specific peaks were only expressed in mixed sex groups. Unfortunately only one of these mating history category-dependent, sex-specific peaks was identified, diethyl succinate in males from mixed sex groups.

Thirty-six compounds had no clear sex bias in either direction; 31 were present in both the virgins and mixed sex groups and five were only present in mixed sex groups. Most of them were intermediate or major in abundance in at least one sex or mating history category. Four, including 2-ethyl-1-hexanol, were in the short-Rt range; one, *N*-2-methylbutyl-2-methylpropanamide, was in the mid-Rt range; and 26, including methyl dodecanoate and ethyl dodecanoate, were in the long-Rt range.

## Discussion

Our use of both SPME GC-MS and GC-FID methods has revealed a previously unknown diversity of volatiles in Qfly rectal glands. It has also identified many of the new compounds, although most of the compounds detected are yet to be identified. Thus, the SPME GC-MS analysis recovered most (24) of the 30 compounds previously identified in organic phase extracts of Qfly rectal glands, and also identified 21 more compounds not previously reported in these glands. Moreover, our GC-FID analyses was then able to detect many further compounds, although without MS data fewer of these could be identified. Still more peaks may have been found had we used additional fibres in the SPME GC-MS analysis, but we expect the number we missed was relatively small. The fibre we used was a broad molecular weight polydimethylsiloxane (PDMS) fibre that has been widely used to analyse tephritid volatiles [24, 30, 55, 82] and Noushini et al. [55] found such a PDMS fibre best able to detect the compounds (including the spiroacetals) that they monitored.

The additional peaks we found were broadly distributed across the short-, mid- and long-Rt ranges but particularly in the latter, with 141 (77%) of all 184 GC-FID peaks analysed in that range. We rated many of the additional peaks as minor (*i*.*e*., < 0.10% of total peak area) in abundance. However, the low percentage abundances of the minor peaks was in large part a consequence of the predominance of the five major amides in males and four of the fatty acid esters in females, which accounted for 87% and 58% of the total peak areas in each sex, respectively.

Of course, a proportion of the peaks we have found may not have any pheromone functions. Some may be associated with other functions of the rectal glands, for example in the final processing steps of frass [83–85]. Others may be incidental occurrences from ingested food or products of their digestion by gut enzymes or microflora [86]. However, the fact that so many of the peaks were not abundant does not preclude the possibility that some of them may have pheromonal functions. Jang et al. [27] found no relationship between abundance and EAG bioactivity among rectal gland volatiles from *C*. *capitata*, and the much studied bombykol of the silk moth *Bombyx mori* is active at as little at 200 molecules per cm^3^ [87]. Notably, some effects of diet on rectal gland volatiles or pheromone compositions have been reported in Qfly and *C*. *capitata* [30, 62, 71] (see also S5 Fig below).

Also consistent with potential pheromone functions for several of the compounds detected is the fact that such functions have already been described for many of the identified compounds in other species [88–90]. Even 2-bornanone, a.k.a. camphor, which is commonly used in ointments and creams and therefore might have been a contaminant, has been recorded in pheromonal secretions in other species, including defensive secretions of the carabid beetle *Carabus lefebvrei* [91] and the ants *Formica japonica* and *Lasius niger* [92], and aggregation pheromones in the larch beetle *Dendroctonus simplex* [93]. (We also note that 2-bornanone was not detected in our blank controls.) The disproportionately high number of short-Rt volatiles that are male-biased or male-specific suggests that some of those compounds could be involved in longer range communication. Conversely, the disproportionately high number of female-biased compounds that are long-Rt suggests that some of those compounds could be involved in shorter range communication. The potential pheromonal function of identified compounds can now be tested in laboratory and field enclosure assays of Qfly behaviours associated with mate attraction and choice [18, 94, 95].

The short-Rt/low KI peaks we identified included five esters, four alcohols, one ketone and two aliphatic aldehydes. Several of these have previously been reported in various fruits, while ethyl propanoate, ethyl 2-methylpropanoate and (*D*, *L*)- and (*meso*)-2,3-butanediol are more widely reported, including in yeasts [58, 96–98]. While this might suggest these compounds were incidental occurrences from ingested food or gut microflora, most of them, in fact, showed a high degree of sex bias. The SPME GC-MS data set included one compound, *x*-octen-1-ol, which was found exclusively in females and seven, including four of the esters, the ketone and two aldehydes, which were found exclusively in males. The GC-FID data set was similar insomuch as it contained two short Rt compounds that were female-biased (and one female-specific), and 19 that were male-biased (16 of them male-specific). Since males and females have similar diets, the high level of specificity evident in both data sets not only suggests endogenous production of the compounds, even if the precursors are food-derived, but also makes them good candidates for specific functions, particularly in males.

The mid-Rt/KI compounds we identified included nine aliphatic amides, six of them previously reported [47, 48, 52, 53, 55, 75] and three of them newly described here. It has been suggested that the six previously described amides are synthesised from two dietary amino acid (leucine and *S-*isoleucine) and three small organic (acetic, propanoic and 2-methylpropanoic) acid precursors [52] (see S4 Fig) and our feeding experiments are consistent with this, insomuch as they show that supplemental *S-*isoleucine enriches for the 2-methylbutyl amides (S5 Fig). The amino acids could be decarboxylated by the corresponding amino acid decarboxylase enzymes, with aminolysis then required to produce the amides. One of the new tentatively identified amides, *N*-(2-methylpropyl)propanamide, might also be produced by such a pathway, in this case from a valine precursor, although the equivalent precursors for the other two, *N*-(*n*-pentyl)propanamide and *N*-(*n*-pentyl)butanamide, are not clear. All six of the previously known amides were classified as major or intermediate in abundance in the GC-FID data set, and five showed the previously reported male bias (the sixth rated as no sex bias). The three new amides were not identified in the GC-FID data set, but one was found in both sexes and the other two just in males in the SPME GC-MS data set. It was earlier proposed, albeit on scant direct evidence, that the amides as a group function as short range male pheromones [45, 48, 52, 54, 75]. One of the other two compounds we identified in the mid-Rt range, diethyl succinate, was only found in males and may be a candidate for such a function.

Most of the identified peaks in the long-Rt/high KI range were fatty acid esters, usually methyl or ethyl but occasionally propyl esters of C12 –C18 fatty acids. The acid groups covered a mix of saturated and (mono) unsaturated carbon chains, and in all but two cases were unbranched. Unsaturation was always at C9 and, with two exceptions, in the 9*Z* (*i*.*e*., *cis*) configuration. The profile of acid groups is thus broadly consistent with those found among the lipids of most organisms [99, 100] and concurs with previous findings from a variety of insects that pheromones are often such fatty acid esters [99–101].

None of the fatty acid esters identified were specific to males; six were found in both sexes and 13 only in females. This predominance in females was also seen in the long-Rt GC-FID peaks overall, and we suggest that many of the unidentified peaks in this range may also be fatty acid esters. Four of the seven fatty acid esters that were identified in the GC-FID data set were rated as major in abundance and the other three were rated as intermediate in abundance. Some of the fatty acid esters may have female-specific functions.

The evidence available suggests that fatty acid esters with pheromone functions in insects are likely derived from lipid pools via oxidation and acetyl transferase reactions on acetyl CoA precursors [99, 100]. These precursors might be produced endogenously or by gut microbes [102–104]. The diversity of lipids/acetyl CoAs potentially available as precursors could account for the large numbers of long-chain esters we have found. Some substrate promiscuity among the relevant redox and transferase enzymes [105–108] could then process the range of acetyl CoAs through to the wide range of esters seen. Some larger alkanes, aldehydes and alcohols implicated in pheromone functions in other insects are also produced in pheromone glands from acetyl CoA starting materials by various redox reactions [109–112]. We identified two long-chain alkanes in our data, and some of the unidentified longer Rt peaks in our data sets may also be larger alkanes, aldehydes and alcohols. Unlike the esters, both the alkanes we identified were only recovered from males, suggesting they may have very different functions from the esters.

We found that the relative abundances of several peaks changed, at least quantitatively and in some cases qualitatively, in their sex bias between the two mating history categories (even though the mixed sex groups could have contained small numbers of virgins). Examples involving identified compounds in the SPME GC-MS data set included ethyl 2-methylbutanoate, which was found in virgins of both sexes but only males in mixed sex groups, and both the butanediols, which were not found in virgins of either sex but were seen in both sexes in mixed sex groups. While it remains to be seen whether these particular cases involve pheromone functions, there are several precedents for effects of mating on pheromone production in other tephritids [24, 113, 114].

One further issue to consider concerns the generality of our findings beyond the particular Qfly stocks used in this study. Our SPME GC-MS results were based on a single laboratory strain, and there is already evidence for some quantitative changes in the abundances of the major amides during domestication [53]. However, our GC-FID results were based on averages of the data from this strain and nine recently collected isofemale lines from various localities across the geographic range of the species (see S4 Table below). While quantitative variation in some peaks was found between the various strains, this did not impact their classification as major, intermediate or minor in abundance [115]. We therefore suggest that our results will be broadly representative of the rectal gland volatile complements of recently collected or domesticated Qfly stocks reared in the laboratory. We are unaware of any reports on the rectal gland volatiles of Qflies collected as adults directly from the field.

In conclusion, our use of high-sensitivity analytical techniques has revealed a diversity of volatiles in Qfly rectal glands, suggesting both a previously underestimated diversity of potential pheromone functions and a complex mix of compounds that could contribute to each function. The diversity is greatest among long-Rt compounds, many of them fatty acid esters, and, against some expectations, predominantly in females. Although the mid-Rt compounds include aliphatic amides, our finding that several of them also occur in females, albeit generally less abundantly than in males, argues against previously proposed male-specific functions for them in mating behaviour. On the other hand, most of the relatively few short-Rt compounds (16 of the 25), which we expect to be highly volatile, were found to be male-specific, and short chain esters, alcohols and a ketone amongst these may be prime candidates for long range male sex pheromone functions. The large number of volatiles in all three Rt categories whose relative abundances differed after mating also raises the possibility of a role in communication of mating status. Key priorities for further work are to test whether rectal gland volatiles showing sex and mating status specificity here can also be detected in headspace analyses of live flies, and then to test those that can be detected in headspace for pheromone functions directly in GC-electroantennogram (GC-EAD) and subsequently behavioural bioassays.

## Materials and methods

### Chemicals

The following compounds were used as authentic standards in the GC-MS and GC-FID assays. Ethyl propanoate (99% purity), 2,3-butanediol isomers (98%), ethyl 2-methylpropanoate (≥ 98%), ethyl 2-methylbutanoate (≥ 98%), *n*-propyl 2-methylpropanoate (≥ 97%), 4-heptanone (98%), 2-ethyl-1-hexanol (≥ 99.6%), diethyl succinate (99%), 2-bornanone (≥ 97%), methyl dodecanoate (≥ 98%), ethyl dodecanoate (≥ 98%), methyl tetradecanoate (≥ 98%), ethyl tetradecanoate (99%), ethyl (*Z*)-9-tetradecenoate (97%), ethyl hexadecanoate (≥ 99%), ethyl (*Z*)-9-octadecenoate (98%) and *n*-hexane (≥ 97%) were procured from Sigma-Aldrich (St. Louis, MO, USA). Ethyl (*Z)*-9-hexadecenoate and ethyl (*E*)-9-octadecenoate, *N*-(2-methylbutyl)acetamide, *N*-(3-methylbutyl)acetamide, *N*-(2-methylbutyl)propanamide, *N*-(3-methylbutyl)propanamide, *N*-(2-methylbutyl)-2-methylpropanamide and *N*-(3-methylbutyl)-2-methylpropanamide, all at ≥ 98.0% purity (by GC), were synthesised previously [53, 55].

### Stocks of flies and rearing

The long-established laboratory stock of Qfly S06 (originally collected from Sydney in 2006 [116]) was used for the SPME GC-MS analysis. This stock, plus nine isofemale lines established from three more recent collections (details in S4 Table), were used for quantitative GC-FID analyses of rectal gland contents. Inclusion of the isofemale lines allowed us to establish the generality of the patterns described herein; comparisons detailed elsewhere [115] showed that a minority of the peaks analysed differed quantitatively among the isofemale lines or between them and S06, but not on a scale affecting the analyses presented here.

### Rectal gland extraction

Mature flies aged 15–20 days after emergence were frozen at -20 ⁰C for three to five days prior to dissection, and rectal glands were excised with entomological forceps by pulling off the terminalia under a stereoscopic microscope (Leica Microsystems, Heidelberg, Germany).

For the SPME GC-MS analysis, ten freshly dissected glands were placed in 10 mL screw-cap SPME headspace vials (Sigma-Aldrich, St. Louis, MO, USA) and stored at -80 ⁰C until analysed as described below. The SPME vials had been incubated at 400°C overnight prior to their use to remove any contaminant volatiles. Replicate samples (generally three), each containing 10 glands in a SPME vial, were prepared for each of the four sex and social categories.

For the GC-FID analysis, triplicate groups of ten to 15 glands of each category, line and generation were individually dissected and collected in 2 mL glass vials (Shimadzu Corporation, Kyoto, Japan) and then immersed in 200 μL high-performance liquid chromatography grade *n*-hexane for 10 min. Subsequently, the hexane was carefully transferred to 2 mL GC vials with 250 μL glass inserts (Shimadzu Corporation). The vials were capped and stored at -80°C until analysed as described below.

### SPME GC-MS data acquisition

SPME GC-MS analyses were performed by electron ionisation at 70 eV on a Shimadzu TQ8050 gas chromatograph-mass spectrometer (Shimadzu Corporation, Kyoto, Japan) fitted with a fused silica DB5 column (30 m x 0.250 mm i.d. x 1 μm film thickness; Agilent Technologies, Santa Clara, USA). Each sample was preincubated for 5 min at 50°C before adsorption into a conditioned 80–500 molecular weight polydimethylsiloxane (PDMS) SPME fibre (30 μm thickness; Restek Corporation, PA, US) for 30 min with constant agitation at 750 rpm. The choice of this fibre was made on the basis of previous studies of tephritid volatiles using SPME [24, 30, 55, 72, 82]. In particular, Noushini et al. [55] found that PDMS fibre performed better on average than PDMS-divinylbenzene and poyacrylate fibres against the range of Qfly esters, amides and spiroacetals they tested. Samples were then desorbed at 250°C for 60 s in the injector fitted with a Topaz SPME inlet liner (Restek Corporation) in a splitless injection mode. Helium was used as carrier gas at a total flow rate of 1 mL/min. Separation involved an initial 40°C oven temperature held for 1 min, increased to 100°C at 10°C/min with 3 min hold and then to 325°C at a rate of 30°C/min with a 10 min final hold. The MS ion source was held at 230°C, and the mass range was set to detect chemicals with m/z values from 20 to 600 (albeit no identification depended on an m/z < 40), with a scan time of 1.0 s and an interscan time of 0.3 s. Empty vials with no rectal glands were used as blanks after every 10 sample injections and at the beginning and end of the analysis sequence.

### GC-FID data acquisition

GC-FID analyses were performed on an Agilent 7890A gas chromatograph (Agilent Technologies) fitted with a HP-5ms UI column (30 m x 0.250 mm i.d. x 0.25 μm film thickness; Agilent Technologies). Sample aliquots (1 μL) were injected using a split mode of 1/10 and helium was used as carrier gas at a constant flow rate of 1 mL/min. The initial oven temperature was programmed at 60°C for 2 min, increased to 100°C at 10°C/min with 3 min hold and then to 325°C at a rate of 5°C/min, with a 10 min final hold at 325°C. The FID detector operated at 300°C. Blank control runs were included as above.

### Data analysis

Data from the blank samples on both instruments were subtracted from the chromatograms to remove peaks that did not originate from the rectal glands. Peaks representing rectal gland chemicals in all chromatograms were manually checked and corrected for retention time shift, following automatic alignment with the GCalignR package in R (version 1.0.2) [117]. The automatic alignment first accounts for systematic shifts in peak retention times among samples, then aligns individual peaks across samples based on variation in retention times across the whole dataset [117]. The maximum accepted linear shift (max_linear_shift) in a peak’s Rt was 0.05 min and the maximum and minimum accepted Rt difference from the peak mean (max_diff_peak2mean and min_diff_peak2mean, respectively) were both 0.03 min. SPME GC-MS analysis for chemical identification was restricted to peaks in a Rt interval from 3 to 23 min.

Tentative identities were assigned to many compounds detected by SPME GC-MS by analysis of their mass spectra and comparisons where possible with those of compounds in the NIST mass spectral library (NIST 14 v2.2), plus, in most cases, comparisons of KI values to published values. The identities of some tentatively identified peaks were then confirmed by matching their retention times to those of authentic standards. Where possible, the identities of GC-FID peaks were assigned by retention time matching against the authentic standards used in the SPME GC-MS.

Peak areas of individual compounds in the GC-FID data were calculated using ACD/Spectrus Processor software version 2019.2.0 (Advanced Chemistry Development Inc., Canada) and were expressed in terms of percentages of total peak areas for statistical analysis.

Qualitative analyses of sex and mating status effects were carried out on each peak that was detected in at least 50% of the samples in at least one of the four sex/mating history categories. Peaks meeting these criteria were then classified as major, intermediate or minor in each category if, on average across the lines included in the analyses, they represented ≥ 1.0%, 0.10–0.99% or < 0.10% of total peak for the category.

Quantitative analyses to investigate differences between sex/mating history categories were also carried out on the GC-FID data using Principal Component Analyses (PCA). Prior to this, each peak was transformed using the best Normalize package (version 1.6.1) [118]. Potential problems with the PCA arising from individual peaks containing multiple compounds were minimal because our use of hexane-extracted glands limited the chemical complexity of our samples.

## Supporting information

S1 Fig Representative SPME GC-MS chromatograms of rectal gland extracts. (**A**) Chromatograms for mixed females (top) and males (bottom); (**B**) Scale-adjusted expansions of different parts of the corresponding Panel A chromatograms labelled with the names of identified compounds. Chromatograms of the samples from mixed sex groups are shown because they contained more of the peaks than the corresponding chromatograms for samples from single sex groups. All identified compounds are named in Panel B, with sex specific compounds in bold.
(DOCX)

S2 Fig GC-MS spectra of five tentatively assigned rectal gland chemicals for which authentic standards and KI were not available. These are: (A) two geometric isomers of *x*-octenal; (B) *x*-octen-1-ol; (C) *N*-(*n*-pentyl)propanamide and D) *N*-(*n*-pentyl)butanamide. As the exact double bond position for octenal isomers and octen-1-ol could not be determined, it is denoted as “x-” and blue colour.
(DOCX)

S3 Fig Representative GC-FID chromatograms of Qfly rectal gland extracts. **(A)** Chromatograms for mixed females (top) and males (bottom); (**B)** Scale-adjusted expansions of different parts of the corresponding Panel **A** chromatograms labelled with the names of identified compounds. Chromatograms of the mixed samples are shown because they contained more of the peaks than the corresponding chromatograms for virgin samples.
(DOCX)

S4 Fig Proposed schema for the production of the six previously identified aliphatic amides from amino and other acids. First amide pair (Amide 1 and 2) are produced from acetic acid, the second pair (Amide 3 and 4) from propanoic acid and the third pair (Amide 5 and 6) from 2-methylpropanoic acid. Amides 1, 3 and 5 are produced from leucine precursors and amides 2, 4 and 6 from isoleucine precursors.
(DOCX)

S5 Fig Relative abundances of the six aliphatic amides in rectal glands of male Qflies fed with isoleucine-supplemented diets. S06 adults were divided in two experimental groups according to the diet provided for the five days before their analysis: A Control group was provided with water and sugar and an Isoleucine group was provided with 10 mM isoleucine in water and sugar. Rectal glands were extracted in hexane analysed by GC-FID as described in the Materials and Methods. The figure shows that for the amide pairs with acetic acid and propanoic acid moieties the amides with isoleucine moieties become relatively more abundant in flies that were fed supplementary isoleucine. The data for the amide pair with 2-methyl propanoic acid moieties were inconclusive because they were difficult to detect on this scale.
(DOCX)

S1 Table Rectal gland chemicals identified in Qfly in this and other studies. The P and C subheadings for *B*. *tryoni* refer to the previous and current studies, respectively. Yellow colour represents females and green colour represent males.
(DOCX)

S2 Table Peaks detected in the CG-FID analysis of the single sex and mixed sex samples categorised by sex specificity and selectivity. Peaks shown as bolded, underlined or plain text are classified as major, intermediate or minor respectively in the category in which they were most abundant. Numbers in each category represent the number of peaks present in each of the three abundance categories, major, intermediate and minor, respectively, divided by commas. “~” refers to peaks with no sex specificity or selectivity. “n.d.” refers to peaks not detected in the corresponding category. Relative abundance is shown in asterisks for the major peaks as follows: *** for compounds above 50%; ** between 20 and 49%, and * for compounds from 1 and 19%.
(DOCX)

S3 Table Peaks found to be major, intermediate, or minor in abundance in each of the four sex/mating history categories. (DOCX)

S4 Table Qfly lines used in the present study. Note that Screened generations refers to the generations in the laboratory post-collection when the rectal gland analyses were carried out.
(DOCX)

S1 Dataset Raw peak areas of peaks detected by GC-FID. The data set contains the peaks that were each present in at least half the samples in at least one of the four sex/mating history categories. The “Cage” column denotes the replicates for each of the lines. The S06 line was also used in the SPME GC-MS analysis. The other nine lines were isofemale lines established from flies collected from various localities around Australia (S4 Table). “Gen” refers to the generation in which the lines were screened. Headings for columns F—GG give the respective retention times (Rts) of the peaks screened.
(XLSX)
