# Correction: Diversity and sex differences in rectal gland volatiles of Queensland fruit fly, *Bactrocera tryoni* (Diptera: Tephritidae)

**DOI:** 10.1371/journal.pone.0317849

**Published:** 2025-01-16

**Authors:** Cynthia Castro-Vargas, Gunjan Pandey, Heng Lin Yeap, Michael J. Lacey, Siu Fai Lee, Soo J. Park, Phillip W. Taylor, John G. Oakeshott

In Table 2, the heading of the columns under the Number of peaks category are incorrect. It should have been "Short-Rt range", "Mid-Rt range", and "Long-Rt range", respectively. Please see the correct Table 2 here.

**Table 2:** Numbers of GC-FID peaks detected classified by sex specificity/ selectivity (F = female, M = male), Rt range and abundance category.

Type	Virgin	Mixed	Number of peaks		
F-biased (34)	F-specific (21)	F-specific (12)	Short Rt range	Mid Rt range	Long Rt range
			0, 0, 1	0, 0, 1	2, 5, 3 **(Ethyl tetradecanoate, 20%)**
		F-selective (3)	-	-	1, 2, 0
					**(Ethyl (*E*)-9-octadecenoate, 5%)**
		~ (2)	-	-	0, 1, 1
		n.d. (4)	0, 1, 0	-	0, 1, 2
	F-selective (10)	F-selective (7)	-	1, 0, 0	**6, 0, 0**
					**(Ethyl (*Z*)-9-hexadecenoate, 23%)**
		~ (2)	-	-	**2, 0, 0**
					**(Ethyl (*Z*)-9-tetradecenoate, 10%)**
		n.d. (1)	-	-	0, 1, 0
	~ (3)	F-specific (2)	-	-	0, 2, 0
		F-selective (1)	-	-	0, 1, 0
					(Methyl tetradecanoate)
M-biased (110)	M-specific (63)	M-specific (49)	0, 6, 4	1, 1, 1	1, 11, 24
			(*n*-propyl 2-methylpropanoate)	**(*N*-(3-Methylbutyl)-2-methylpropanamide, 5%)**	
		n.d. (10)	0, 0, 1	1, 0, 0	0, 1, 7
		~ (4)	-	-	0, 0, 4
	M-selective (4)	M-specific (2)	-	0, 2, 0	-
				(*N*-(2-Methylbutyl)acetamide)	
		M-selective (2)	-	2, 0, 0	-
				**(*N*-(3-Methylbutyl)acetamide, 9%)**	
				**(*N*-(3-Methylbutyl)propanamide, 67%)**	
	~ (14)	M-specific (12)	0, 0, 3 (4-Heptanone^#^)	0, 0, 1	1, 2, 5
		M-selective (2)	-	1, 0, 0	1, 0, 0
				**(*N*-(2-Methylbutyl)propanamide, 4%)**	
	n.d. (29)	M-specific (29)	0, 1, 4	0, 1, 4	0, 4, 15
				(Diethyl succinate)	
Changed specificity (4)	M-specific (2)	F-specific (2)	-	-	0, 2, 0
	F-specific (2)	M-specific (2)	-	-	0, 0, 2
No sex bias (36)	~ (31)	~ (31)	0, 4, 0	1, 0, 0	1, 22, 3
			(2-Ethyl-1-hexanol^#^)	**(*N*-(2-Methylbutyl)-2-methylpropanamide, 2%)**	(Methyl dodecanoate)
					(Ethyl dodecanoate)
	n.d. (5)	~ (5)	-	-	0, 4, 1

The numbers in brackets in the three left-hand columns are the total numbers of peaks in the respective sex/mating history categories. The three numbers in each cell in the three right-hand columns are the numbers of peaks classified as major, intermediate and minor in abundance, respectively. The names of identified peaks in those cells are given in brackets and are bolded, underlined or in plain text if major, intermediate or minor in abundance respectively in the sex/mating history combination in which they were most abundant. For the major identified peaks, their % of summed peak area in the sex/mating history combination in which they were most abundant is also given in the brackets. “~” refers to peaks with no sex specificity or selectivity. Within each mating history category, sex-specific peaks were only detected in one sex; sex-selective peaks showed > log_10_ differences in average peak areas between the sexes; and those with no strong sex bias showed < log_10_ differences in average peak areas between the sexes. “n.d.” refers to peaks not detected in the corresponding category. “^#^” refers to peaks not identified in Qfly rectal glands in previous studies.

In S2 Table, the heading of the columns under the Peaks category are incorrect. It should have been "Short-Rt range", "Mid-Rt range", and "Long-Rt range", respectively. Please view the correct S2 Table below.

In the GC-FID profiles subsection of Results, there is an error in the second sentence of the third paragraph. The correct sentence is: These Rt ranges in our data are approximately equivalent to KI ranges of 840–1095, 1096–1240 and 1241–2175, respectively.

## Supporting information

S1 Table Peaks detected in the CG-FID analysis of the single sex and mixed sex samples categorised by sex specificity and selectivity. Peaks shown as bolded, underlined or plain text are classified as major, intermediate or minor respectively in the category in which they were most abundant. Numbers in each category represent the number of peaks present in each of the three abundance categories, major, intermediate and minor, respectively, divided by commas. “~” refers to peaks with no sex specificity or selectivity. “n.d.” refers to peaks not detected in the corresponding category. Relative abundance is shown in asterisks for the major peaks as follows: *** for compounds above 50%; ** between 20 and 49%, and * for compounds from 1 and 19%.
(DOCX)

## References

[1] Castro-Vargas C, Pandey G, Yeap HL, Lacey MJ, Lee SF, Park SJ, et al. (2022) Diversity and sex differences in rectal gland volatiles of Queensland fruit fly, *Bactrocera tryoni* (Diptera: Tephritidae). PLoS ONE 17(8): e0273210. doi: 10.1371/journal.pone.0273210 36001616 PMC9401129

