# Supplementary figures and images for: Diversity and sex differences in rectal gland volatiles of Queensland fruit fly, Bactrocera tryoni (Diptera: Tephritidae)
Source: PLoS One. 2022 Aug 24;17(8):e0273210. doi: 10.1371/journal.pone.0273210 (PMC9401129; doi:10.1371/journal.pone.0273210)

**S1 Fig**


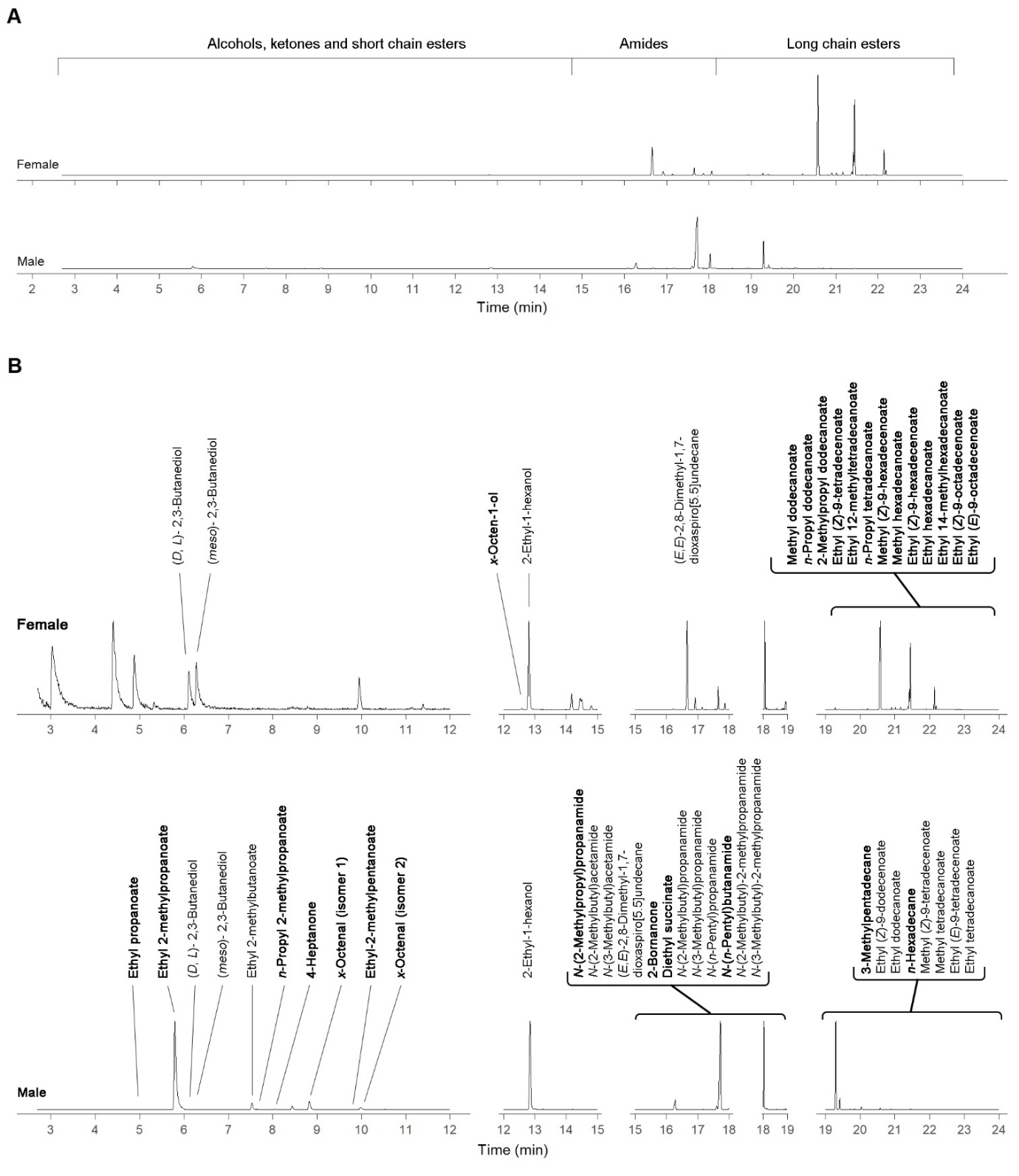

Supplement: S1 Fig — (A) Chromatograms for mixed females (top) and males (bottom); (B) Scale-adjusted expansions of different parts of the corresponding Panel A chromatograms labelled with the names of identified compounds. Chromatograms of the samples from mixed sex groups are shown because they contained more of the peaks than the corresponding chromatograms for samples from single sex groups. All identified compounds are named in Panel B, with sex specific compounds in bold. (DOCX) [file pone.0273210.s001.docx]

**S2 Fig**

**A) *x*-Octenal isomers**

**
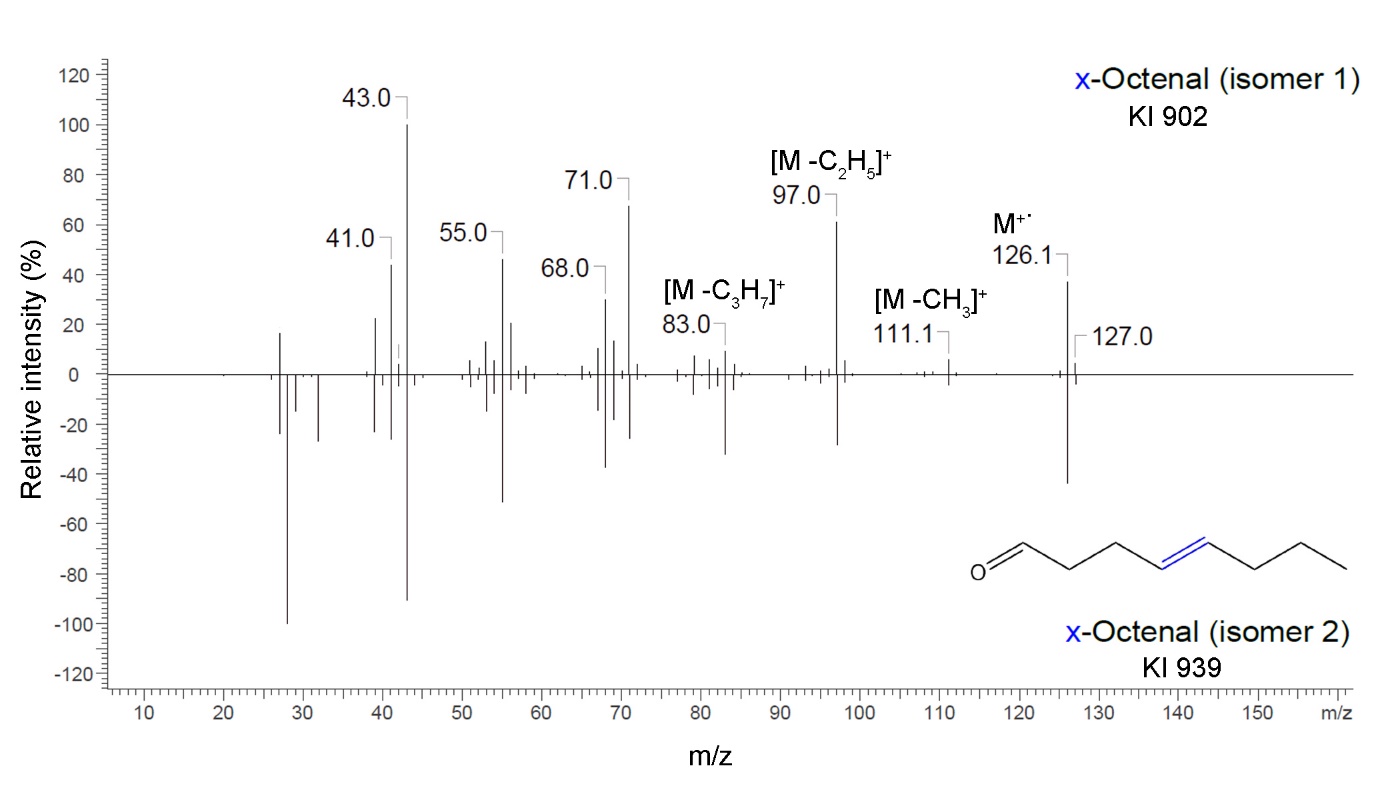
**

**B) *x*-Octen-1-ol**

**
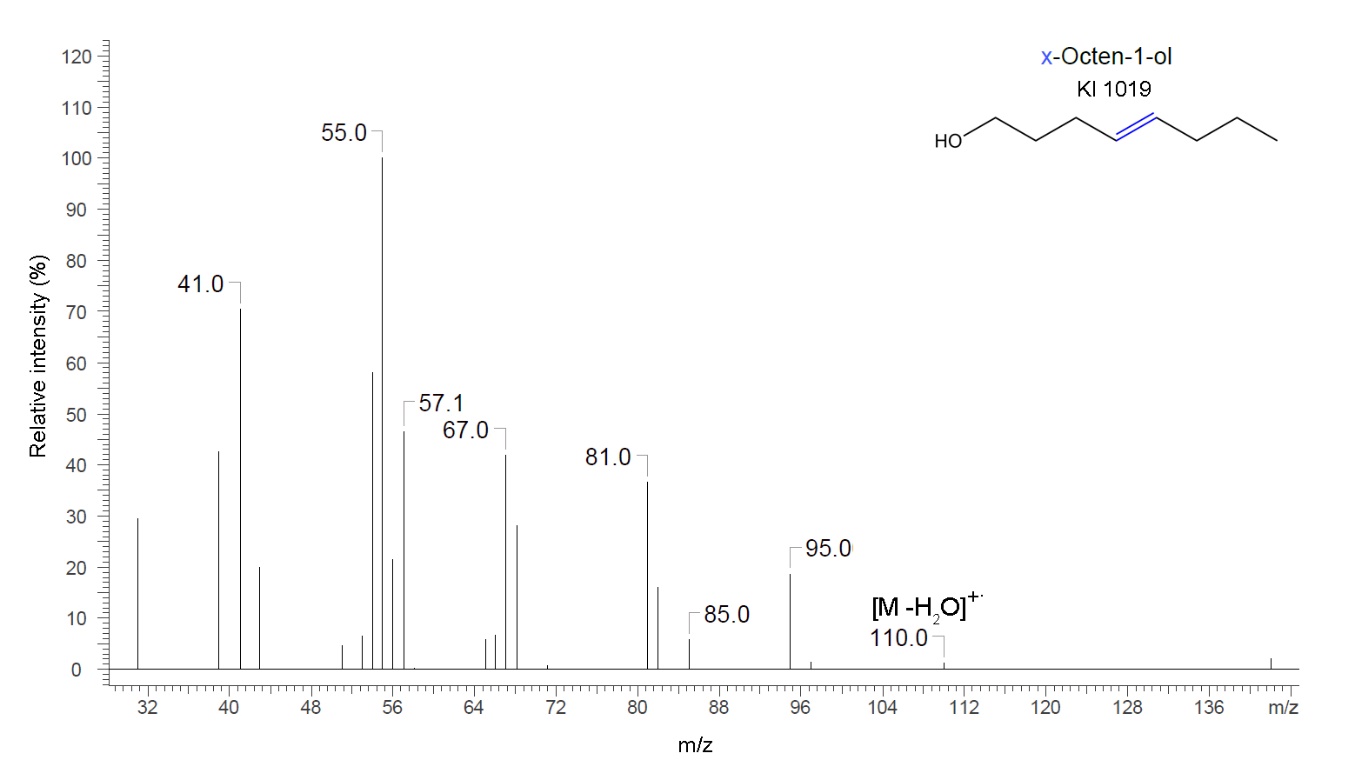
**

**C)** ***N-(n*-Pentyl)propanamide**

**
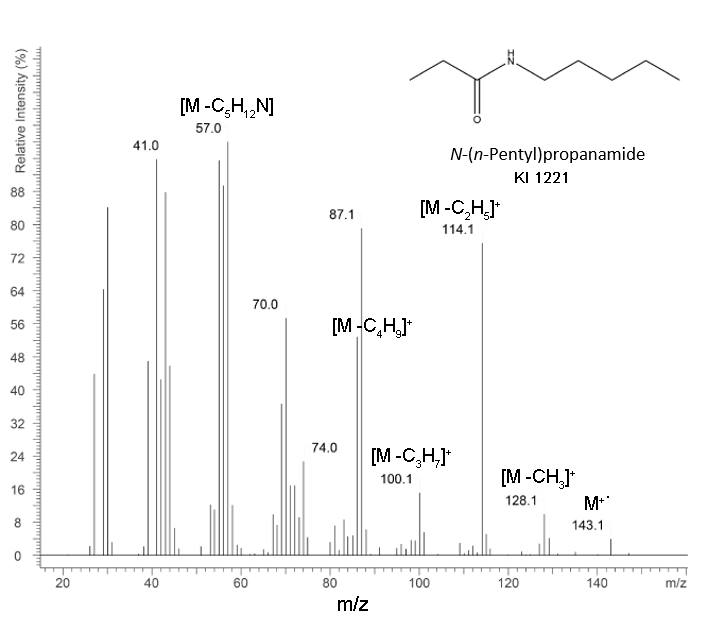
**

**D) *N*-(*n*-Pentyl)butanamide**


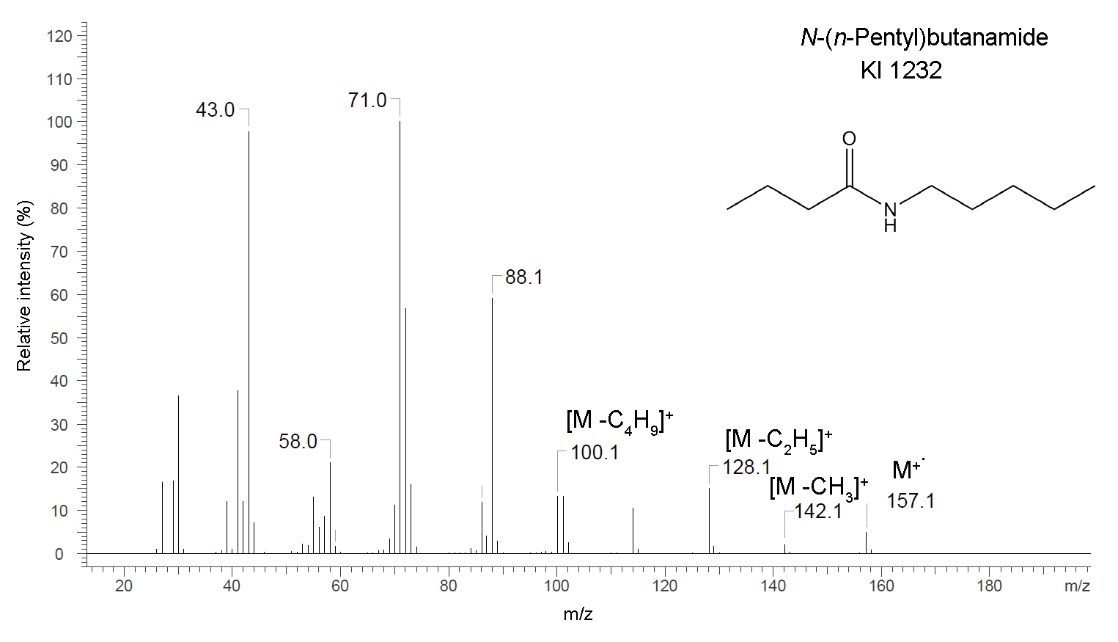

Supplement: S2 Fig — These are: (A) two geometric isomers of x-octenal; (B) x-octen-1-ol; (C) N-(n-pentyl)propanamide and D) N-(n-pentyl)butanamide. As the exact double bond position for octenal isomers and octen-1-ol could not be determined, it is denoted as “x-” and blue colour. (DOCX) [file pone.0273210.s002.docx]

**S3 Fig**

**
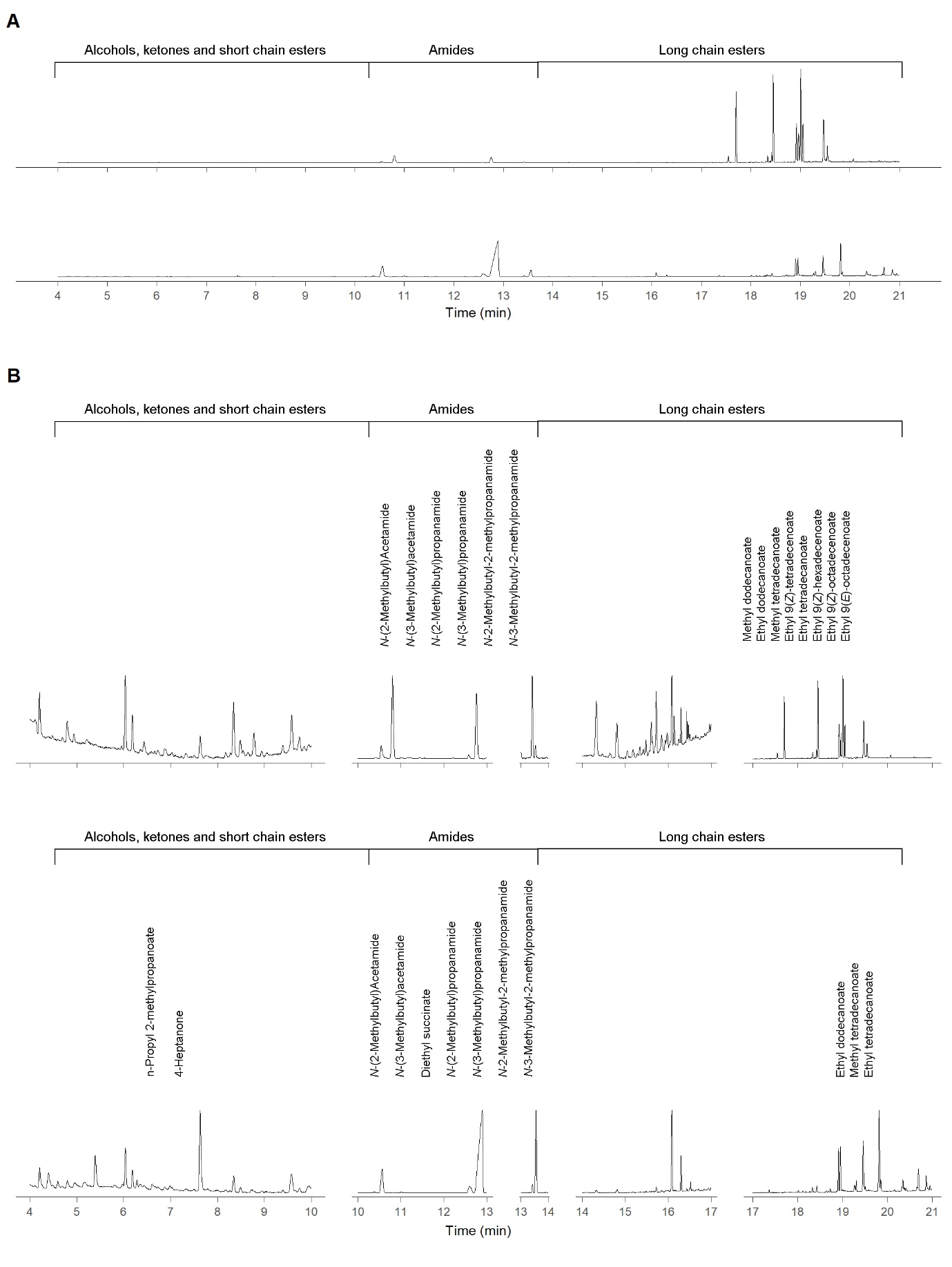
**

Supplement: S3 Fig — (A) Chromatograms for mixed females (top) and males (bottom); (B) Scale-adjusted expansions of different parts of the corresponding Panel A chromatograms labelled with the names of identified compounds. Chromatograms of the mixed samples are shown because they contained more of the peaks than the corresponding chromatograms for virgin samples. (DOCX) [file pone.0273210.s003.docx]

**S4 Fig**

**
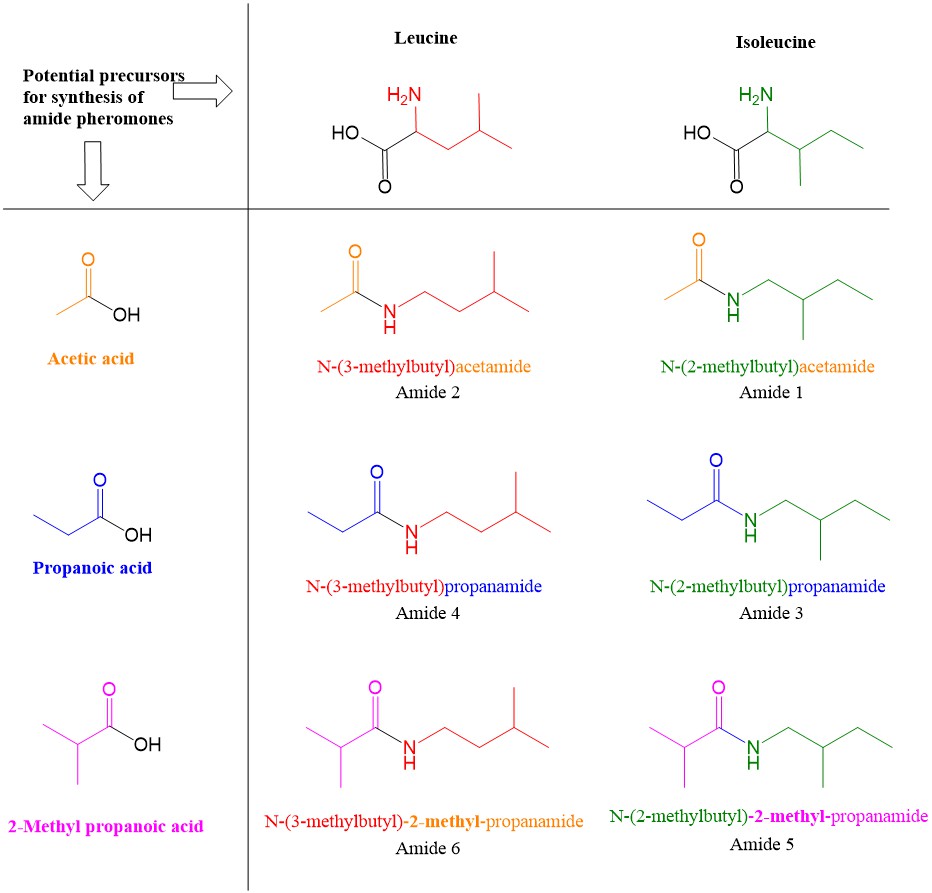
**

Supplement: S4 Fig — First amide pair (Amide 1 and 2) are produced from acetic acid, the second pair (Amide 3 and 4) from propanoic acid and the third pair (Amide 5 and 6) from 2-methylpropanoic acid. Amides 1, 3 and 5 are produced from leucine precursors and amides 2, 4 and 6 from isoleucine precursors. (DOCX) [file pone.0273210.s004.docx]

**S5 Fig**

**
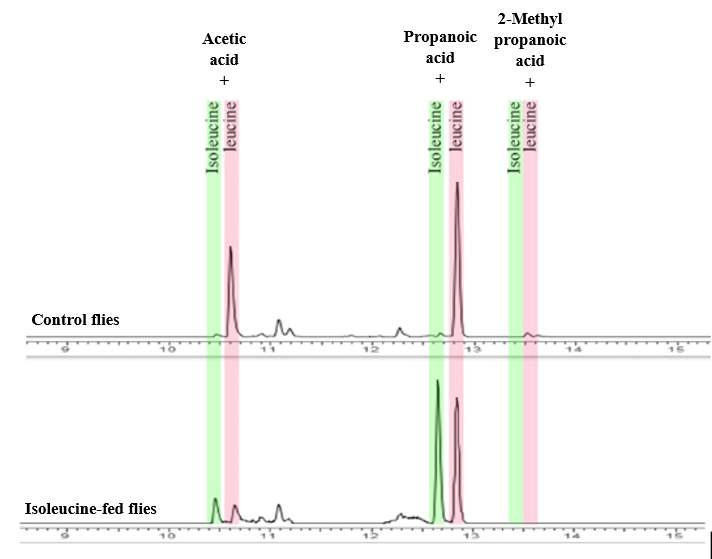
**

Supplement: S5 Fig — S06 adults were divided in two experimental groups according to the diet provided for the five days before their analysis: A Control group was provided with water and sugar and an Isoleucine group was provided with 10 mM isoleucine in water and sugar. Rectal glands were extracted in hexane analysed by GC-FID as described in the Materials and Methods. The figure shows that for the amide pairs with acetic acid and propanoic acid moieties the amides with isoleucine moieties become relatively more abundant in flies that were fed supplementary isoleucine. The data for the amide pair with 2-methyl propanoic acid moieties were inconclusive because they were difficult to detect on this scale. (DOCX) [file pone.0273210.s005.docx]
