# Supplementary material for: Diversity and sex differences in rectal gland volatiles of Queensland fruit fly, Bactrocera tryoni (Diptera: Tephritidae)
Source: PLoS One. 2022 Aug 24;17(8):e0273210. doi: 10.1371/journal.pone.0273210 (PMC9401129; doi:10.1371/journal.pone.0273210)
Supplement: S1 Table — The P and C subheadings for B. tryoni refer to the previous and current studies, respectively. Yellow colour represents females and green colour represent males. (DOCX) [file pone.0273210.s006.docx]

**S1 Table**

| **Semiochemical** | P | C | **Semiochemical** | P | C |
| --- | --- | --- | --- | --- | --- |
| ***Alcohols*** | | | ***Esters (cont.)*** | | |
| (*D*,*L*)-2,3-Butanediol |  | **⚫⚫** | Ethyl (*Z*)-9-tetradecenoate | **⚫** | **⚫** |
| (*meso*)-2,3-Butanediol |  | **⚫⚫** | Ethyl dodecanoate | **⚫** | **⚫⚫** |
| 2-Ethyl-1-hexanol |  | **⚫⚫** | Ethyl hexadecanoate | **⚫** | **⚫** |
| *x*-Octen-1-ol |  | **⚫** | Ethyl propanoate | **⚫** | **⚫** |
| ***Aldehydes*** |  |  | Ethyl tetradecanoate | **⚫** | **⚫⚫** |
| *x*-Octenal (isomers) |  | **⚫** | 2-Methylpropyl dodecanoate |  | **⚫** |
| ***Amides*** |  |  | Methyl (*Z*)-9-hexadecenoate | **⚫** | **⚫** |
| *N*-(2-Methylbutyl)acetamide | **⚫⚫** | **⚫⚫** | Methyl (*Z*)-9-tetradecenoate |  | **⚫⚫** |
| *N*-(2-Methylbutyl)propanamide | **⚫⚫** | **⚫⚫** | Methyl dodecanoate | **⚫** | **⚫** |
| *N*-(2-Methylpropyl)propanamide |  | **⚫** | Methyl hexadecanoate |  | **⚫** |
| *N*-(3-Methylbutyl)acetamide | **⚫⚫** | **⚫⚫** | Methyl tetradecanoate | **⚫** | **⚫⚫** |
| *N*-(3-Methylbutyl)propanamide | **⚫⚫** | **⚫⚫** | *n*-Propyl dodecanoate | **⚫** | **⚫** |
| *N*-(2-Methylbutyl)-2-methylpropanamide | **⚫** | **⚫⚫** | *n*-Propyl 2-methylpropanoate | **⚫** | **⚫** |
| *N*-(3-Methylbutyl)-2-methylpropanamide | **⚫⚫** | **⚫⚫** | *n*-Propyl tetradecanoate |  | **⚫** |
| *N*-hexyl propanamide | **⚫** |  | ***Ketones*** |  |  |
| *N*-propyl butanamide | **⚫** |  | 4-Heptanone |  | **⚫** |
| *N*-(*n-*Pentyl)propanamide |  | **⚫⚫** | ***Spiroacetals*** |  |  |
| *N*-(*n-*Pentyl)butanamide |  | **⚫** | (*E*,*E*)-2,8-Dimethyl-1,7-dioxaspiro[5.5]undecane | **⚫** | **⚫⚫** |
| ***Esters*** |  |  | (*E*,*E*)-2-Ethyl-2,8-dimethyl-1,7-dioxaspiro[5.5]undecane | **⚫** |  |
| Diethyl succinate | **⚫** | **⚫** | (*E*,*E*)-2-Ethyl-8-methyl-1,7-dioxaspiro[5.5]undecane | **⚫** |  |
| Ethyl 12-methyltetradecanoate |  | **⚫** | (*E*,*E*)-2-Propyl-8-methyl-1,7-dioxaspiro[5.5]undecane | **⚫** |  |
| Ethyl 14-methylhexadecanoate |  | **⚫** | (*E*,*Z*)-2-Ethyl-7-methyl-1,6-dioxaspiro[4.5]decane | **⚫** |  |
| Ethyl 2-methylbutanoate | **⚫** | **⚫⚫** | 2,7-Dimethyl-1,6-dioxaspiro[4.5]decane | **⚫** |  |
| Ethyl 2-methylpentanoate | **⚫** | **⚫** | ***Terpenes*** |  |  |
| Ethyl 2-methylpropanoate | **⚫** | **⚫** | 2-Bornanone |  | **⚫** |
| Ethyl (*E*)-9-octadecenoate | **⚫** | **⚫** | ***Others*** |  |  |
| Ethyl (*E*)-9-tetradecenoate |  | **⚫⚫** | 3-Methylpentadecane |  | **⚫** |
| Ethyl (*Z*)-9-dodecenoate |  | **⚫⚫** | *n*-Hexadecane |  | **⚫** |
| Ethyl (*Z*)-9-hexadecenoate | **⚫** | **⚫** |  |  |  |
| Ethyl (*Z*)-9-octadecenoate | **⚫** | **⚫** |  |  |  |
