# Supplementary material for: Diversity and sex differences in rectal gland volatiles of Queensland fruit fly, Bactrocera tryoni (Diptera: Tephritidae)
Source: PLoS One. 2022 Aug 24;17(8):e0273210. doi: 10.1371/journal.pone.0273210 (PMC9401129; doi:10.1371/journal.pone.0273210)
Supplement: S2 Table — Peaks shown as bolded, underlined or plain text are classified as major, intermediate or minor respectively in the category in which they were most abundant. Numbers in each category represent the number of peaks present in each of the three abundance categories, major, intermediate and minor, respectively, divided by commas. “~” refers to peaks with no sex specificity or selectivity. “n.d.” refers to peaks not detected in the corresponding category. Relative abundance is shown in asterisks for the major peaks as follows: *** for compounds above 50%; ** between 20 and 49%, and * for compounds from 1 and 19%. (DOCX) [file pone.0273210.s007.docx]

**S2 Table**

| **Type** | **Single sex** | **Mixed sex** | **Peak (Rt)** | | |
| --- | --- | --- | --- | --- | --- |
| Female biased  (34) | Female specific (21) | Female specific (12) | KI range 848 – 1130 | KI range 1130 – 1239 | KI range 1239 – |
|  |  |  | 8.82 | 11.27 | 15.51, **18.40*, 18.46 (Ethyl tetradecanoate)****, 18.73, 18.78, 18.83, 19.59, 20.05, 20.11, 20.25 |
|  |  | Female selective (3) |  |  | 19.14, **19.51 (Ethyl (*E*)-9-octadecenoate)***, 20.55 |
|  |  | ~ (2) |  |  | 18.61, 19.76 |
|  |  | n.d. (4) | 9.63 |  | 18.85, 19.70, 19.99 |
|  | Female selective (10) | Female selective (7) |  | **10.85*** | **17.55*, 17.69**, 18.88*, 18.98 (Ethyl (*Z*)-9-hexadecenoate)**, 19.01*, 19.39*** |
|  |  | ~ (2) |  |  | **18.42 (Ethyl (*Z*)-9-tetradecenoate)**, 18.93*** |
|  |  | n.d. (1) |  |  | 20.52 |
|  | ~ (3) | Female specific (2) |  |  | 18.37, 20.31 |
|  |  | Female selective (1) |  |  | 18.21 (Methyl tetradecanoate) |
| Male biased  (110) | Male specific (63) | Male specific (49) | 4.61 (*n*-propyl 2-methylpropanoate), 5.20, 5.41, 5.99, 6.30, 6.64, 6.94, 8.53, 9.43, 9.58 | 11.46, 11.93, **13.59 (*N*-(3-Methylbutyl)-2-methylpropanamide)*** | 13.79, 13.95, 15.22, 15.38, 15.67, 15.98, 15.99, 16.18, 16.52, 16.59, 16.66, 16.85, 16.89, 16.94, 17.01, 17.07, 17.17, 17.26, 17.41, 17.64, 17.75, 17.84, 18.01, 18.48, 18.68, 18.80, **18.89***, 19.08, 19.12, 19.62, 19.72, 19.83, 20.33, 20.59, 20.64, 20.98 |
|  |  | n.d. (10) | 10.05 | **13.33*** | 16.73, 17.72, 18.76, 19.04, 19.65, 20.19, 20.35, 20.81 |
|  |  | ~ (4) |  |  | 15.59, 18.72, 20.01, 20.13 |
|  | Male selective (4) | Male specific (2) |  | 10.41 (*N*-(2-Methylbutyl)acetamide), 11.04 |  |
|  |  | Male selective (2) |  | **10.59 (*N*-(3-Methylbutyl)acetamide )*, 12.96 (*N*-(3-Methylbutyl)propanamide)***** |  |
|  | ~ (14) | Male specific (12) | 4.82 (4-Heptanone), 4.96, 6.21 | 12.27 | 15.87, 17.15, 17.32, 17.52, 18.12, 18.53, **19.48***, 20.07 |
|  |  | Male selective (2) |  | **12.63 (*N*-(2-Methylbutyl)propanamide)*** | **19.43*** |
|  | n.d. (29) | Male specific (29) | 4.07, 7.79, 8.73, 9.79, 9.98 | 11.7, 11.79 (Diethyl succinate),11.97, 12.45, 13.34 | 13.88, 14.54, 15.07, 15.45, 16.14, 16.70, 16.78, 18.02, 19.15, 19.20, 19.31, 19.78, 19.92, 19.94, 19.97, 20.22, 20.23, 20.40, 20.42 |
| Changed specificity (4) | Male specific (2) | Female specific (2) |  |  | 18.39, 20.62 |
|  | Female specific (2) | Male specific (2) |  |  | 15.63, 20.15 |
| No sex bias  (36) | ~ (31) | ~ (31) | 4.23, 6.06, 7.66 (2-Ethyl-1-hexanol), 8.38 | **13.47 (*N*-(2-Methylbutyl)-2-methylpropanamide)*** | 14.37, 14.86, 15.77, **16.11***, 16.32, 16.45, 17.21, 17.37 (Methyl dodecanoate), 17.45, 17.63 (Ethyl dodecanoate), 17.92, 17.96, 18.10, 18.17, 18.27, 18.32, 18.64, 19.25, 19.29, 19.35, 19.56, 19.66, 19.80, 19.86, 20.50, 20.84 |
|  | n.d. (5) | ~ (5) |  |  | 16.50, 20.37, 20.68, 20.88, 20.90 |
