# Supplementary material for: Diversity and sex differences in rectal gland volatiles of Queensland fruit fly, Bactrocera tryoni (Diptera: Tephritidae)
Source: PLoS One. 2022 Aug 24;17(8):e0273210. doi: 10.1371/journal.pone.0273210 (PMC9401129; doi:10.1371/journal.pone.0273210)
Supplement: S3 Table — (DOCX) [file pone.0273210.s008.docx]

**S3 Table**

| **Virgin males** | **Mixed males** | **Virgin females** | **Mixed females** |
| --- | --- | --- | --- |
| MAJOR | | | |
| 10.59 (8.8%), 12.63 (3.6%), 12.96 (67.2%), 13.33 (1.8%), 13.47 (1.9%), 13.59 (3.7%), 16.11 (1.4%) | 10.59 (8.8%), 12.63 (2.2%), 12.96 (54.6%), 13.59 (5.2%), 16.11 (1.6%), 18.89 (2.9%), 18.93 (2.7%), 19.43 (3%), 19.48 (1.5%) | 10.59 (1.8%), 10.85 (5%), 12.96 (11.1%), 17.69 (13.6%), 18.40 (1.4%), 18.42 (10.2%), 18.46 (9.5%), 18.88 (2.5%), 18.93 (6.6%), 18.98 (15.3%), 19.01 (6%), 19.39 (2.3%), 19.51 (3.5%) | 10.85 (3.1%), 12.96 (2.9%), 17.55 (1.0%), 17.69 (14.8%), 18.40 (1.3%), 18.42 (1.2%), 18.46 (20.1%), 18.88 (4.4%), 18.93 (2.3%), 18.98 (22.6%), 19.01 (8.5%), 19.39 (4.7%), 19.51 (4.5%) |
| INTERMEDIATE | | | |
| 4.23, 5.41, 7.66, 8.38, 9.58, 10.41, 11.04, 14.86, 15.77, 16.32, 16.94, 17.21, 17.32, 17.45, 18.01, 18.32, 18.39, 18.42, 18.64, 18.89, 18.93, 18.98, 19.25, 19.43, 19.56, 19.80, 19.83, 20.31, 20.59, 20.62, 20.64, 20.81, 20.84 | 5.20, 5.41, 5.99, 6.06, 6.64, 7.66, 9.58, 9.98, 10.41, 11.04, 11.46, 13.34, 13.47, 14.37, 16.32, 16.94, 17.37, 17.63, 17.64, 17.69, 17.75, 17.84, 17.92, 17.96, 18.01, 18.02, 18.12, 18.27, 18.32, 18.42, 18.64, 18.68, 18.98, 19.01, 19.08, 19.29, 19.31, 19.51, 19.56, 19.80, 19.83, 20.33, 20.37, 20.40, 20.42, 20.64, 20.68, 20.84, 20.88, 20.90, 20.98 | 4.23, 7.66, 8.38, 9.63, 11.04, 12.63, 13.47, 14.86, 15.77, 16.11, 16.45, 17.21, 17.32, 17.45, 17.55, 17.96, 18.10, 18.17, 18.21, 18.32, 18.37, 18.64, 18.73, 18.78, 18.83, 18.85, 19.14, 19.25, 19.29, 19.43, 19.48, 19.56, 19.80, 20.05, 20.07, 20.11, 20.52, 20.55 | 7.66, 10.59, 12.63, 13.47, 16.11, 17.96, 18.10, 18.21, 18.32, 18.37, 18.39, 18.61, 18.64, 18.73, 18.78, 18.83, 19.14, 19.25, 19.29, 19.35, 19.43, 19.56, 19.80, 20.05, 20.31, 20.55, 20.62 |
| MINOR | | | |
| 4.61, 4.82, 4.96, 5.20, 5.99, 6.06, 6.21, 6.30, 6.64, 6.94, 8.53, 9.43, 10.05, 10.85, 11.46, 11.93, 12.27, 13.79, 13.95, 14.37, 15.22, 15.38, 15.59, 15.67, 15.87, 15.98, 15.99, 16.18, 16.45, 16.52, 16.59, 16.66, 16.73, 16.85, 16.89, 17.01, 17.07, 17.15, 17.17, 17.26, 17.37, 17.41, 17.52, 17.55, 17.63, 17.64, 17.69, 17.72, 17.75, 17.84, 17.92, 17.96, 18.10, 18.12, 18.17, 18.21, 18.27, 18.37, 18.48, 18.53, 18.68, 18.72, 18.76, 18.80, 18.88, 19.01, 19.04, 19.08, 19.12, 19.29, 19.35, 19.39, 19.48, 19.62, 19.65, 19.66, 19.72, 19.86, 20.01, 20.07, 20.13, 20.19, 20.33, 20.35, 20.50, 20.52, 20.98 | 4.07 4.23, 4.61, 4.82, 4.96, 6.21, 6.30, 6.94, 7.79, 8.38, 8.53, 8.73, 9.43, 9.79, 10.85, 11.70, 11.79, 11.93, 11.97, 12.27, 12.45, 13.79, 13.88, 13.95, 14.54, 14.86, 15.07, 15.22, 15.38, 15.45, 15.59, 15.63, 15.67, 15.77, 15.87, 15.98, 15.99, 16.14, 16.18, 16.45, 16.50, 16.52, 16.59, 16.66, 16.70, 16.78, 16.85, 16.89, 17.01, 17.07, 17.15, 17.17, 17.21, 17.26, 17.32, 17.41, 17.45, 17.52, 17.55, 18.10, 18.17, 18.21, 18.48, 18.53, 18.61, 18.72, 18.80, 18.88, 19.12, 19.14, 19.15, 19.20, 19.25, 19.35, 19.39, 19.62, 19.66, 19.72, 19.76, 19.78, 19.86, 19.92, 19.94, 19.97, 20.01, 20.07, 20.13, 20.15, 20.22, 20.23, 20.50, 20.55, 20.59 | 4.82, 4.96, 6.06, 6.21, 8.82, 10.41, 11.27, 12.27, 14.37, 15.51, 15.63, 15.87, 16.32, 17.15, 17.37, 17.52, 17.63, 17.92, 18.12, 18.27, 18.53, 18.61, 19.35, 19.59, 19.66, 19.70, 19.76, 19.86, 19.99, 20.15, 20.25, 20.31, 20.50, 20.84 | 4.23, 6.06, 8.38, 8.82, 11.27, 14.37, 14.86, 15.51, 15.59, 15.77, 16.32, 16.45, 16.50, 17.21, 17.37, 17.45, 17.63, 17.92, 18.17, 18.27, 18.72, 19.59, 19.66, 19.76, 19.86, 20.01, 20.11, 20.13, 20.25, 20.37, 20.50, 20.68, 20.84, 20.88, 20.90 |
