# Supplementary material for: Diversity and sex differences in rectal gland volatiles of Queensland fruit fly, Bactrocera tryoni (Diptera: Tephritidae)
Source: PLoS One. 2022 Aug 24;17(8):e0273210. doi: 10.1371/journal.pone.0273210 (PMC9401129; doi:10.1371/journal.pone.0273210)
Supplement: S4 Table — Note that Screened generations refers to the generations in the laboratory post-collection when the rectal gland analyses were carried out. (DOCX) [file pone.0273210.s009.docx]

**S4 Table**

| **Set** | **Population** | **Line** | **Location** | | **Screened generations** | **Collection date** |
| --- | --- | --- | --- | --- | --- | --- |
|  |  |  | **Latitude** | **Longitude** |  |  |
| Isofemale lines | Alice Springs | AS09, AS19, AS36 | -23.69 | 133.89 | 6,7,8,10 | Nov 2017 |
|  | Cape Tribulation | CT07, CT38, CT60 |  |  | 4,5,6,8 | Aug 2018 |
|  | Sydney | SY13, SY18, SY53 | -33.90 | 151.14 | 6,7,8,10 | Sep 2017 |
| Mass-bred laboratory strain | Sydney | S06 |  |  | >120 | 2006 |
